# Assimilation of L2 vowels to L1 phonemes governs L2 learning in adulthood: a behavioral and ERP study

**DOI:** 10.3389/fnhum.2014.00279

**Published:** 2014-05-14

**Authors:** Mirko Grimaldi, Bianca Sisinni, Barbara Gili Fivela, Sara Invitto, Donatella Resta, Paavo Alku, Elvira Brattico

**Affiliations:** ^1^Dipartimento di Studi Umanistici, Centro di Ricerca Interdisciplinare sul Linguaggio, Università del SalentoLecce, Italy; ^2^Laboratorio di Anatomia Umana e Neuroscience, Dipartimento di Scienze e Tecnologie Biologiche e Ambientali, Università del SalentoLecce, Italy; ^3^Department of Signal Processing and Acoustics, Aalto UniversityEspoo, Finland; ^4^Brain & Mind Laboratory, Department of Biomedical Engineering and Computational Science, Aalto UniversityEspoo, Finland; ^5^Cognitive Brain Research Unit, Institute of Behavioral Sciences, University of HelsinkiHelsinki, Finland

**Keywords:** adult phoneme perception, mismatch negativity (MMN), foreign language acquisition, L2 classroom learning, event-related potentials, vowel perception

## Abstract

According to the Perceptual Assimilation Model (PAM), articulatory similarity/dissimilarity between sounds of the second language (L2) and the native language (L1) governs L2 learnability in adulthood and predicts L2 sound perception by naïve listeners. We performed behavioral and neurophysiological experiments on two groups of university students at the first and fifth years of the English language curriculum and on a group of naïve listeners. Categorization and discrimination tests, as well as the mismatch negativity (MMN) brain response to L2 sound changes, showed that the discriminatory capabilities of the students did not significantly differ from those of the naïve subjects. In line with the PAM model, we extend the findings of previous behavioral studies showing that, at the neural level, classroom instruction in adulthood relies on assimilation of L2 vowels to L1 phoneme categories and does not trigger improvement in L2 phonetic discrimination. Implications for L2 classroom teaching practices are discussed.

## Introduction

Learning a second language (L2) in adulthood challenges our brains. As mother tongue phoneme representations are formed in the brains of 6–12 months old children (Werker and Tees, 1983; Kuhl et al., 1992; Cheour et al., 1998; Kuhl, 2008) non-native speech sounds become increasingly difficult to discriminate and L2 perception generally turns into a demanding task for learners (Iverson et al., 2003). This loss of sensitivity does not prevent L2 learning in adulthood (Flege, 1995). The extent of success may depend nonetheless on numerous variables: i.e., age of L2 learning, length of residence in an L2-speaking country, gender, formal instruction, motivation, language learning aptitude and amount of native language (L1) use (see Piske et al., 2001 for an overview). When L2 learners are immersed in an L2 environment, the contribution of age toward learning to perceive and produce L2 sounds occurs primarily through interactions with the amount of L1 use and the amount of L2 native speaker input received (Flege et al., 1995, 1997, 1999; Flege and Liu, 2001; Flege and MacKay, 2004; Tsukada et al., 2005; see Piske, 2007 for a critical review). However, when learners are immersed in an L1 environment and have a reduced L2 exposure, primarily in a restricted setting (namely, with little or unsystematic conversational experience with native speakers) learning of L2 phonemes at the native speaker level becomes very difficult if not impossible. According to Best and Tyler (2007: 16), the perception of L2 in these individuals receiving only formal instruction in adulthood may resemble that of L2 naïve listeners. In other words, they are functional monolinguals, not actively learning or using L2 when compared with L2-learning listeners, i.e., learners who are in the process of actively learning an L2 to achieve functional, communicative goals within natural L2 context.

Cross-linguistic and L2 speech perception studies have shown that adult learners of L2 have difficulty with both the perception and production of non-native phonological segments, i.e., consonants and vowels that either do not occur or are phonetically different in their L1 (see Flege, 2003 for a discussion). Indeed, it is commonly thought that a major determinant of L2 foreign accent is the underlying problem associated with the perception of L2 phonological structures. In turn, acquisition of phonetic contrasts involves not only the detection of differences in the acoustic signal but also the accessing of internalized categories, which in the brain are most likely associated with definite neural representations. Within the behavioral literature, there are two major theoretical frameworks on L2 speech learning in adulthood, the Speech Learning Model (SLM, Flege, 1995) and the Perceptual Assimilation Model (PAM, Best, 1995). The SLM has been primarily concerned with the ultimate attainment of L2 production and perception and mainly deals with highly experienced L2 learners immersed in an L2 environment, whereas the PAM is mainly interested in explaining the initial L2 perception of L2 learners through the non-native perception of naïve listeners, who are in fact functional monolinguals (but see Best and Tyler, 2007, for an extension to L2 learning). Both SLM and PAM posit that the degree of success listeners will have in perceiving non-native L2 sounds depends on the perceived relationship between phonetic elements found in the L1 and the L2 systems. These models make predictions about performance in non-native segmental perception based on the perceived distance between L1 and L2 sounds (Guion et al., 2000).

This study investigated the thus far little studied L2 perception in functional monolinguals, by behaviorally and neurally testing the predictions posed by the PAM framework. The PAM predicts that if two non-native sounds are perceived as acceptable exemplars of two distinct native phonemes (Two-Category assimilation), their discrimination will be easy, while if both non-native sounds are perceived to be equally poor/good exemplars of the same native phoneme (Single-Category assimilation), their discrimination will be difficult. An intermediate discrimination is predicted when the two non-native sounds are both perceived as the same native sound but differ in goodness rating (Category-Goodness assimilation). Finally, when an L2 category is perceived as more than one L1 phoneme and the other L2 category is perceived as a single native phoneme, a good discrimination is predicted (Uncategorized-Categorized assimilation). For predictions to be generated by PAM (or the SLM), cross-language phonetic distance data need to be obtained by means of behavioral experiments. The degree of perceptual distance between phonemes is usually examined using an identification and rating methodology. The foreign (or L2) sounds are first classified as instances of a phonetic category(s) in the listener's L1, then rated for goodness-of-fit to the L1 category.

Whereas the studies on L2 and non-native phoneme perception discussed above have used only behavioral techniques to address this question, we chose to adopt both behavioral (categorization and discrimination tests) and electrophysiological (event-related potential, ERP) techniques to examine the L2 perceptual abilities of our subjects. The ERP technique provides not only a millisecond precise measurement of information processing in the brain but also, depending upon the task, can allow one to disentangle automatic detection from attentional processes. ERP studies on L2 phoneme processing have used the oddball paradigm, alternating repetitive (standard) and infrequent (deviant) sounds (80–20% of occurrence respectively) while subjects are distracted from listening by a primary task (e.g., watching a silent movie), to measure the so-called mismatch negativity (MMN) response to L2 contrasts. The MMN is an ERP component, elicited by stimulus change at ≈100–250 ms, mainly generated in the auditory cortex and with additional generators in the inferior frontal cortex, reflecting the neural detection of a change in a constant property of the auditory environment (Picton et al., 2000; Näätänen et al., 2007). A large body of evidence supports the notion that the discriminative MMN process relies both on auditory sensory and categorical phonetic representations of speech stimuli and that these two codes are utilized in parallel by the pre-attentive change detection process reflected in the MMN component (Näätänen et al., 2001, 2011; Pulvermüller and Shtyrov, 2006). The MMN results from prediction violations on the basis of the repetitive standard presentation (Winkler and Czigler, 2012). It has been proposed that the standard presentation resembles perceptual learning during which hierarchical sensory levels of processing receive bottom-up sensory input from lower levels and receive top-down predictions from higher levels (Garrido et al., 2009). As a result of the repetition of the standard presentation, prediction errors are reduced by repetitive suppression or adaptation (Friston, 2005). A deviant presentation then leads to a violation of bottom-up prediction that is reflected in MMN generation (see also the discussion in Scharinger et al., 2012). Furthermore, the amplitude and peak latency of the MMN is directly correlated with the magnitude of the perceived change and, hence, it is considered a measure of individual discrimination accuracy (see Amenedo and Escera, 2000; Näätänen, 2001; Sussman et al., 2013 for a critical discussion).

The results of MMN studies, mainly focused on L2-learning listeners, are mixed. For instance, Winkler et al. (1999a) found that Hungarian adult late L2 learners who had been immersed for several years in the L2 context perceived non-native contrasts (in Finnish) as well as native speakers, as evidenced by comparable MMN amplitudes elicited by both native Finns and fluent Hungarians in response to a Finnish across category-boundary vowel contrast, when opposed to naïve Hungarians. The results by Winkler et al. (1999a) were not replicated in a population of advanced adult L2 learners (of English) who were not immersed, since advanced Finnish students of English did not show MMN to English phonemes that would be comparable to the one elicited by native Finnish phonemes, hence suggesting that learning in the classroom environment may not lead to the formation of new long-term native-like memory traces (Peltola et al., 2003). These brain responses to new phonemes probably develop in children at a very fast pace: i.e., within three months of intensive exposure, as evidenced by MMN to L2 phoneme contrasts in Finnish children participating in French language immersion education (Cheour et al., 2002; Shestakova et al., 2003; Peltola et al., 2005). Again, however, subsequent works did not confirm these findings when the L2 was English both for Finnish listeners (Peltola et al., 2007) and Japanese listeners (Bomba et al., 2011). Finally, Rinker et al. (2010) for bilingual Turkish–German kindergarten children growing up in Germany have shown that the MMN response is less robust in Turkish–German children to the German vowel, when compared to a German control group. Thus, immersion education and natural acquisition contexts did not guarantee native-like L2 vowel discrimination. Also, native-like L2 vowel discrimination is not guaranteed after a short training (50 min on 5 consecutive days) via associative/statistical learning: as showed by Dobel et al. (2009), who neurally investigates the perceptual acquisition of an L2 consonant (/ϕ/) in a group of adult German speakers using the MEG methodology. Instead of establishing a novel category the subjects integrated /ϕ/ into the native category /f/, demonstrating that native categories are powerful attractors hampering the mastery of non-native contrasts. None of these studies, though, have tried to explain the L2 perceptual processes according to any of the well-established models for L2 learning. Hence they left open the question of which mechanisms govern the acquisition of L2 phonemes in adult learners from formal instruction and with restricted L2 exposure.

The present study aims at studying the behavioral and neural (MMN) correlates of L2 learning in adulthood while directly testing the hypotheses that these correlates would index the perceptual mechanisms posed by the PAM model. Specifically, our study addressed two questions: (i) Do the predictions generated by the PAM through behavioral methods hold when they are neurophysiologically investigated, namely can the discrimination patterns predicted by the PAM for L2 naïve listeners be also mirrored in MMN amplitudes or latencies? (ii) Is L2 classroom learning associated with the typology of L2 naïve listeners, as recently suggested by Best and Tyler (2007)? To answer these questions, we measured the behavioral and electrophysiological data of two groups of Salento Italian (SI) undergraduate students of British English (BE) attending the first and the fifth year of the Foreign Languages and Literatures Faculty. Crucially, SI, the Italian variety spoken in Southern Apulia, presents a five stressed vowel system (i.e., /i, ε, a, ͻ, u/; Grimaldi, 2009; Grimaldi et al., 2010) contrary to the richer vowel system of BE that shows, excluding diphthongs, eleven stressed vowels (see Stimuli). Therefore, for SI speakers, it could be relatively difficult to learn a complex L2 vowel system, supporting the idea that the L1 plays an important role and enables one to predict the relative difficulty of acquisition of a given L2 contrast (Iverson and Evans, 2007). Firstly, we behaviorally tested the two groups of students by means of an identification test. On the basis of the results of this test, the contrasts /iː/-/uː/ and /æ/-/Λ/ (for which the PAM's framework predicted an excellent and a good discrimination, respectively) were selected for a behavioral discrimination test. In the ERPs experiment, the groups of students were compared with a control group of listeners who were much more linguistically inexperienced of the L2, as their knowledge of English derived only from compulsory school studies. Moreover, as a control condition we introduced the L1 within-category contrast /ε/-[e], for which poor discrimination is predicted (cf. Phillips et al., 1995; Dehaene-Lambertz, 1997; Winkler et al., 1999b; see also Miglietta et al., 2013). These two vowels are phonologically contrastive in standard Italian and they are used to create lexical contrast (i.e., /‵pεska/ “peach” vs. /‵peska/ “fishing”) whereas SI has the phoneme /ε/ only. Consequently, for SI speakers these stimuli belong to the same category, as /ε/ is the underlying phoneme and [e] represents an *allophone* (generally transcribed between brackets), namely a within-category variant of the same phoneme.

## Methods

### Behavioral experiments

#### Subjects

Two groups of 10 normal-hearing (tested prior to the experiment), right-handed, undergraduate male students of the Foreign Languages and Literatures Faculty voluntarily participated in the experiments. One group was enrolled in its first year (age 21.4 ± 1.71; 9.4 ± 1.34 years of English studies in formal context), whereas the other was in its fifth year (age 25.6 ± 1.98; 14.3 ± 2.11 years of English studies in formal context). As assessed by a questionnaire of language use, all the subjects neither participated in Erasmus programs in England nor have had L2 native teachers prior to attending university. English instruction university classes are taught by Italian native-speakers prevalently, although for at least 6 months per year (3–5 h per week) these students had been attending lessons also with native English lecturers. However, in the last case, language classes are only a few hours per week and are just based on lexical and morphosyntactic formal instructions; no systematic and explicit phonetic instruction or training is administered.

#### Stimuli

The stimuli consisted of the 11 BE monophthong vowels, i.e., /iː/, /I/, /ε/, /æ/, /Λ/, /ɑː/, /ɒ/, /зː/, /ͻː/, /℧/ and /uː/ (Ladefoged, 2001). These sounds were produced by three male native BE speakers (age 47.3 ± 4.9; years in Italy: 22.3 ± 5.13), two of them coming from London, one coming from Birmingham. The speakers read a list of monosyllabic words with the phonemes /iː/, /I/, /ε/, /æ/, /Λ/, /ɑː/, /ɒ/ and /зː/ placed in a /p_t/ context and the phonemes /iː/, /ͻː/, /℧/ and /uː/ in an /s_t/ context, for a total of 36 stimuli (3 speakers × 12 phonemes). Given that /iː/-/uː/ and /uː/-/℧/ were part of the discrimination task as control and target contrasts, respectively, /iː, ℧ and uː/ needed to be recorded in the same consonant context. Thus, the extra context /s_t/ was used for these three vowels because there is no English word with /uː/ in the /p_t/ context. These stimuli were recorded in the CRIL soundproof room by a CSL 4500 at a sampling rate of 22.05 kHz and were segmented and normalized in peak amplitude using the software Praat 4.2. Each of the student groups performed two perceptual tests: the identification and the oddity discrimination test. All subjects were individually tested in the CRIL soundproof room using a computer and with sounds (set at a comfortable sound level) delivered via headphones, for a total duration of approximately 40 min.

#### Identification test

The aim of the identification test was to examine the perceived phonetic distance between the L1 and L2 sounds: i.e., to detect which L2 sounds are more similar/dissimilar to the L1 sounds and, consequently, are more difficult/easy to discriminate by perception (Flege and MacKay, 2004). The 36 stimuli were randomly presented 3 times, and subjects identified each of them in terms of one of the 5 SI vowels /i, ε, a, ͻ/ or /u/ by clicking on the computer screen. Students could not rehear a stimulus, but they were told to guess if they were unsure. Before performing the test, students received instructions orally and a training test of 10 stimuli was administered in the presence of the experimenter to ensure that the students understood the task. No subject was rejected on the basis of the training test because they all found the task easy to perform.

#### Oddity discrimination test

The purpose of the oddity discrimination test was to measure the ability of listeners to discriminate L2 sounds. For each of the two contrasts, 8 change trials and 8 catch trials (32 total trials per student) were executed. The change trials were made up of 3 items, each one produced by one of the three BE speakers, with an odd item belonging to a different phonological category that subjects had to detect. The odd item was alternatively placed in the first, second or third position in a nearly balanced way (Tsukada et al., 2005) to avoid response bias (Bion et al., 2006). Additionally, the three native English speakers produced the catch trials, where all of the items contained the same phonological category. These kinds of trials test subjects' ability to ignore the acoustical differences among the stimuli belonging to the same phonological category. For instance, to test the contrast /iː/-/uː/ the change trials were /iː/-/iː/-/uː/ − /iː/-/uː/-/iː/ − /uː/-/iː/-/iː/ − /uː/-/uː/-/iː/ − /uː/-/iː/-/uː/ − /iː/-/uː/-/uː/, and the catch trials were /iː/-/iː/-/iː/ − /uː/-/uː/-/uː/. Subjects clicked the computer screen on “1,” “2,” “3,” corresponding to the position of the item they perceived as different or to “none” if they perceived all items as equal. The results of this test, i.e., A' scores, were calculated for each contrast by applying the formula of Snodgrass et al. (1985). These scores reduce the effects of response bias by calculating the proportion of hits (i.e., the number of correct selections of the odd item in the change trials) and the proportion of false alarms (i.e., the number of incorrect selections of an odd item in the catch trials). An A' score of 1.0 indicates perfect discrimination and an A' score of 0.5 indicates a null discrimination. Subjects were first given the instructions and then administered a training test in the presence of the experimenter to verify that they had understood the task. No subject was rejected on the basis of the training test because they all found the task easy to perform. This test was also executed by a control group of 10 male BE listeners (mean age: 20.5 ± 1.95), native speakers of the London variety.

***Statistical analysis of oddity discrimination test results.*** Discrimination accuracy (A' score) was analyzed in repeated-measures ANOVA with “contrast” (/æ/-/Λ/ and /iː/-/uː/) as the within-subject factor and “group” (first and fifth year) as the between-subject factor. In all of the statistical analyses, the alpha level was set to *p* < 0.05, and type I errors were controlled for by decreasing the degrees of freedom with the Greenhouse–Geisser epsilon. *Post-hoc* tests were conducted by Fisher's least-significant difference (LSD) comparisons.

### ERP experiment

#### Subjects

The two groups of students involved in the behavioral experiments participated in the ERP sessions. Additionally, a third control group of normally hearing (tested prior to the experiment), right-handed subjects with only compulsory school education (10 subjects; age 25 ± 4.26; years of English studies in formal context 5 ± 2.9) performed the electrophysiological test. The control group was primarily composed of carpenters, plasterers, or unemployed, and each participant received a small monetary compensation for participating in the experiment. If one considers that in Italy a foreign language is usually taught starting from the last two years of primary school (when children are normally 8 years old), we can suppose that the student groups and the control group have a similar starting age of L2 exposure. However, the student groups have more formal exposure to the L2, particularly the fifth year group. In contrast, the control group's L2 exposure was limited to compulsory school, where they passively received impoverished lexical or morphosyntactic inputs by non-native L2 teachers for approximately 3 h per week. Additionally, in Italy foreign programs are dubbed, so that the exposure to foreign languages in informal contexts is very low. We also excluded that the ordinary listening of English music could represent an involuntary L2 training, as the acquisition of L2 in adulthood presupposes a strong motivation and a continuous use of L2 in different conversational contexts (cf. Gardner, 1991). All of the subjects signed the informed consent form. The local Ethics Committee approved the experimental procedure.

#### Stimuli and procedure

We used the same contrast pairs as in the oddity discrimination test but the stimuli consisted of synthetic vowels whose duration was 350 ms (edited with Praat 4.2). Thus the contrasts tested were /i/-/u/ and /æ/-/Λ/. A third contrast was added as control, i.e., /ε/-[e] where the former is a mid-opened vowel and the latter a mid-closed one. This is a within-category contrast for SI speakers and poor discrimination is predicted. In Table 1, we provide the acoustic characteristics of stimuli. First formant frequency (F1) and second formant frequency (F2) are given in Hz.

**Table 1:** **Values of the first formant (F1) and the second formant (F2) given in Hz and Euclidean distances of the stimulus contrasts utilized in the ERP experiment**.

**Formants**	**/i/**	**/u/**	**/æ/**	**/Λ/**	**/ε/**	**[e]**	**Contrast**	**Euclidean distance**
F1 Hz	322	347	823	678	563	435	/i/-/u/	1209 Mel
F2 Hz	2363	1015	1535	1090	1712	1986	/æ/-/Λ/	581 Mel
							/ε/-[e]	404 Mel

To avoid confounding the effects of acoustic variations in natural utterances with the ERP responses, the stimuli for the ERP experiment were created using the Semisynthetic Speech Generation method (SSG, Alku et al., 1999), which mathematically models the functioning of the human voice production mechanism. To obtain raw material for the SSG synthesis for the ERP experiment, short words produced by a native male BE speaker (44 years old coming from London) and by a native male speaker of Standard Italian (45 years old, coming from Florence) were recorded in a soundproof room using a Sennheiser MKH 20 P48 high-frequency condenser, omnidirectional microphone, and a response frequency of 20–20,000 Hz, and further processed with a sampling frequency of 22050 Hz and a resolution of 16 bits. Signal sections corresponding to the desired vowels to be synthesized were cut from the recorded words. From these selected sections, the corresponding vocal tract filters were computed with SSG using digital all-pole filtering (Oppenheim and Schafer, 1989) of 22.

The three contrasts /æ/-/Λ/, /i/-/u/ and /ε/-[e] were presented in separate blocks lasting 15 min each, and each with 86% frequency of occurrence (582 trials) for the standard stimulus (the first vowel of each above listed pair) and 14% frequency (114 trials) for the deviant stimulus (the second vowel of each pair). The order of presentation was pseudo-randomized, since a deviant stimulus was never presented before three standards. The interstimulus interval was 750 ms. During the EEG recording, participants sat in a comfortable armchair and were instructed to watch a silent movie while paying no attention to the stimuli, which were binaurally presented in a soundproof room through loudspeakers at 65/70 dB.

#### Electrophysiological recordings

The EEG was recorded from the scalp using a 64 Ag/AgCl electrode cap (BrainCap, Brain Products) with a sampling frequency of 500 Hz. Eye movements were monitored with electrodes attached at the top and the bottom of the left eye and at the top of the right eye. The reference electrodes were attached on the ear lobes. Impedance was kept under 15 kΩ. The signal was off-line filtered (0.5–50 Hz, 24 dB), and the threshold for artifact rejection was set at > ±125 μ V. The numbers of trials accepted after artifact rejection are reported in Table 2. Each standard following a deviant was removed from the averaging. The ERP epochs included a pre-stimulus interval of 100 ms, used for baseline correction, and lasted until 450 ms.

**Table 2:** **The average number of accepted standard (stand) and deviant (dev) trials for each contrast and each group (control group, first year students, fifth year students)**.

**Contrasts**	**Control**		**First year**		**Fifth year**	
	**stand**	**dev**	**stand**	**dev**	**stand**	**dev**
/æ/-/Λ/	496 (85%)	97 (85%)	510 (88%)	99 (87%)	491 (85%)	98 (86%)
/i/-/u/	472 (86%)	93 (86%)	500 (86%)	98 (86%)	512 (88%)	101 (89%)
/ε/-[e]	501 (86%)	98 (86%)	495 (85%)	99 (86%)	491 (84%)	98 (86%)

The percentages with respect to the total number of trials are also given in parentheses.

#### Statistical analysis of ERP data

To quantify the MMN, we first identified the most negative peaks at Fz around the time interval 120–300 ms for each contrast and group from the grand-average difference waveforms. Subsequently, the individual MMN amplitudes were calculated by taking the mean values from the same 40-ms interval around the grand-average MMN peaks for each contrast and group obtained as described above. The significance of the individual MMN amplitudes at Fz was verified by paired *t*-tests against the zero baseline. To test our hypotheses on the effects of contrast types and language exposure on the MMN amplitudes measured at F3, F4, C3, C4, P3, and P4, we used repeated-measures ANOVAs and linear mixed-effect models with the between-subject factor Group (first year, fifth year students and control group) and the within-factors Language (the within-category contrast /ε/-[e] and the English pairs /i/-/u/ and /æ/-/Λ/), Contrast (/i/-/u/, /æ/-/Λ/, and /ε/-[e]), Frontality (frontal, central, and parietal electrodes) and Laterality (right or left hemisphere). We also extracted the individual peak latencies of the MMN response recorded at Fz by searching for the most negative peak within the time interval 120–300 ms per each subject and each condition. For testing the hypotheses on the MMN peak latencies, a similar ANOVA as above (with Group, Language and Contrast as factors) was conducted but without the two electrode factors. For all statistical tests, the alpha level was chosen to correspond to *p* < 0.05. Type I errors were controlled for by decreasing the degrees of freedom with the Greenhouse–Geisser epsilon (original degrees of freedom are reported) or by adding subjects as random effect including it as intercept or random slopes, when appropriate as assessed by the Bayesian information criteria in a linear mixed-effect model. The difference threshold for accepting or rejecting a more complex model was set to 4. *Post-hoc* tests were conducted by Fisher's least-significant difference (LSD) comparisons.

## Results

### Identification test

The identification test results were considered in terms of the percentage of identification of BE phonemes with respect to the SI ones. The percentages indicate the frequency with which L1 SI vowels were used to classify the L2 BE vowels. The percentages of identification obtained by first (I) and fifth (V) year students are summarized in Table 3.

**Table 3:**
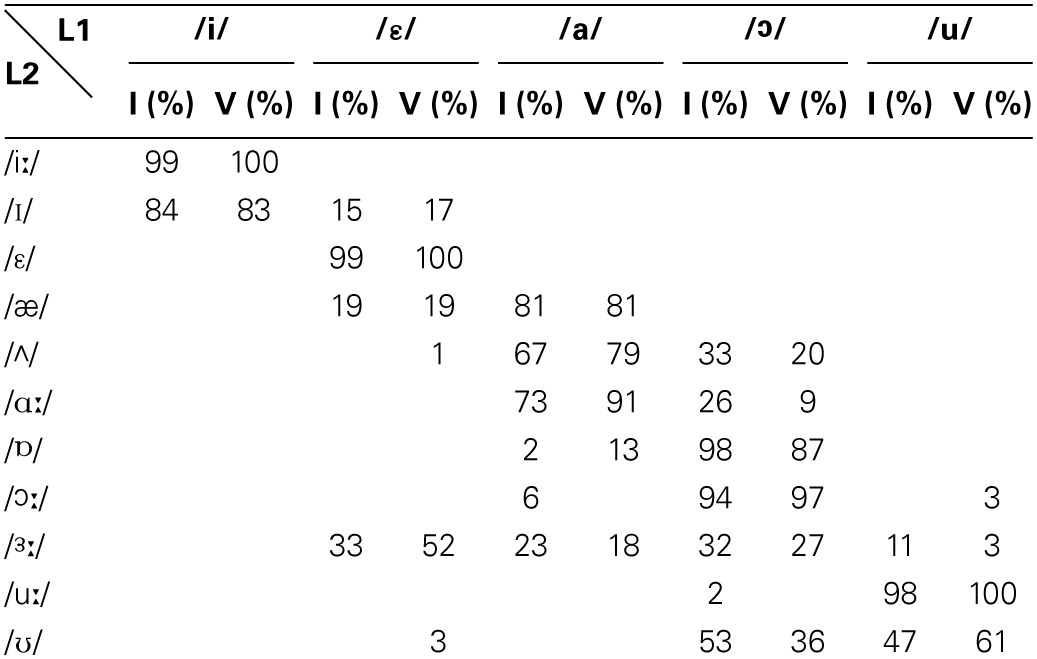
**Mean percentage of identification of L2 vs. L1 vowels by first (I) and fifth (V) year students**.

The percentages of identification of the L2 phonemes to the L1 phonemes are very useful for understanding how the former are perceived and categorized with respect to the latter. The L2 phonemes associated with an L1 phoneme with an identification percentage ≥ 80% were considered consistently identified to the L1 and only that identification was taken into account. Conversely, those L2 phonemes associated with two or more L1 phonemes (identification percentage < 80%) were considered as not consistently assimilated, and the first two identifications were taken into account.

The data summarized in Table 3 show that both the first and the fifth year students adopted the same assimilation strategies, albeit with slightly different percentages. According to the identification consistency threshold identified above, the results depict the following scenario: /æ/ was consistently assimilated with the native phoneme /a/; /Λ/ was identified to /a/ or /o/, so was not assimilated to either of these two native phonemes. Finally, /iː/ and /uː/ were each consistently identified with the native phonemes, /i/ and /u/, respectively. In fact, BE /iː/ and /uː/ (see Table 1) share some formant features with SI /i/ (F1 326, F2 2244) and /u/ (F1 368, F2 867) (Grimaldi, 2009) and consequently are perceived by SI listeners as their native counterpart.

According to the PAM typologies of assimilation, the vowels /æ/, /Λ/, /iː/ and /uː/, can be grouped into two contrasts of L2 vowels (see Table 3): (i) the contrast /æ/-/Λ/ falls into the Uncategorized-Categorized assimilation, for which good discrimination is predicted, as the non-native vowel /æ/ is consistently assimilated to a native phoneme (/a/), whereas the other vowel /Λ/ is not categorized with any native phoneme; (ii) the contrast /iː/-/uː/ falls into the two-category assimilation, for which excellent discrimination is predicted, as they have been consistently identified with two different native phonemes: i.e., /i/ and /u/. The discrimination ability by the two groups of students for these contrasts was further tested with the oddity discrimination test.

### Oddity discrimination test

**Figure 1:**
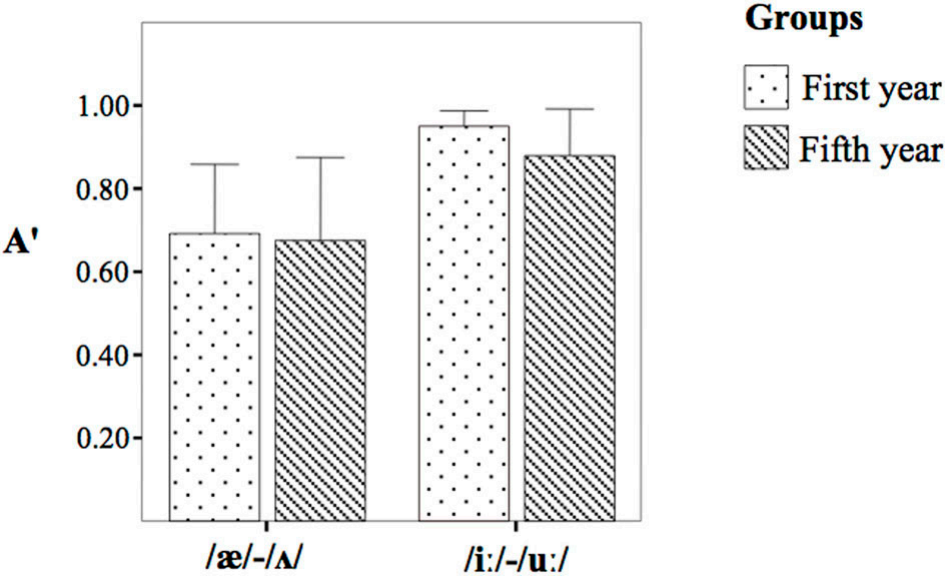
**The A' score obtained by the first year group (dotted bar) and the fifth year group (striped bar)**.

The repeated-measures ANOVA on A' scores (Table 4 and Figure 1) did not yield differences between the two groups, [*F*_(1, 18)_ = 0.40, *p* > 0.05, η^2^_*p*_ = 0.02] but it yielded a significant effect for the contrasts [*F*_(1, 18)_ = 18.24, *p* = 0.000, η^2^_*p*_ = 0.50]. The *post-hoc* analysis revealed that the contrast /iː/-/uː/ was discriminated with a higher A' with regard to the contrast /æ/-/Λ/. The interaction Group × Contrast was not significant [*F*_(1, 18)_ = 0.26, *p* > 0.05, η^2^_*p*_ = 0.01].

**Table 4:** **The A' scores obtained by the first year group (I) and the fifth year group (V)**.

**Contrasts**	**I year group**	**V year group**
/æ/-/Λ/	0.69 (0.23)	0.67 (0.27)
/iː/-/uː/	0.95 (0.04)	0.87 (0.15)

Standard deviations are in parentheses.

### ERPs

**Figure 2:**
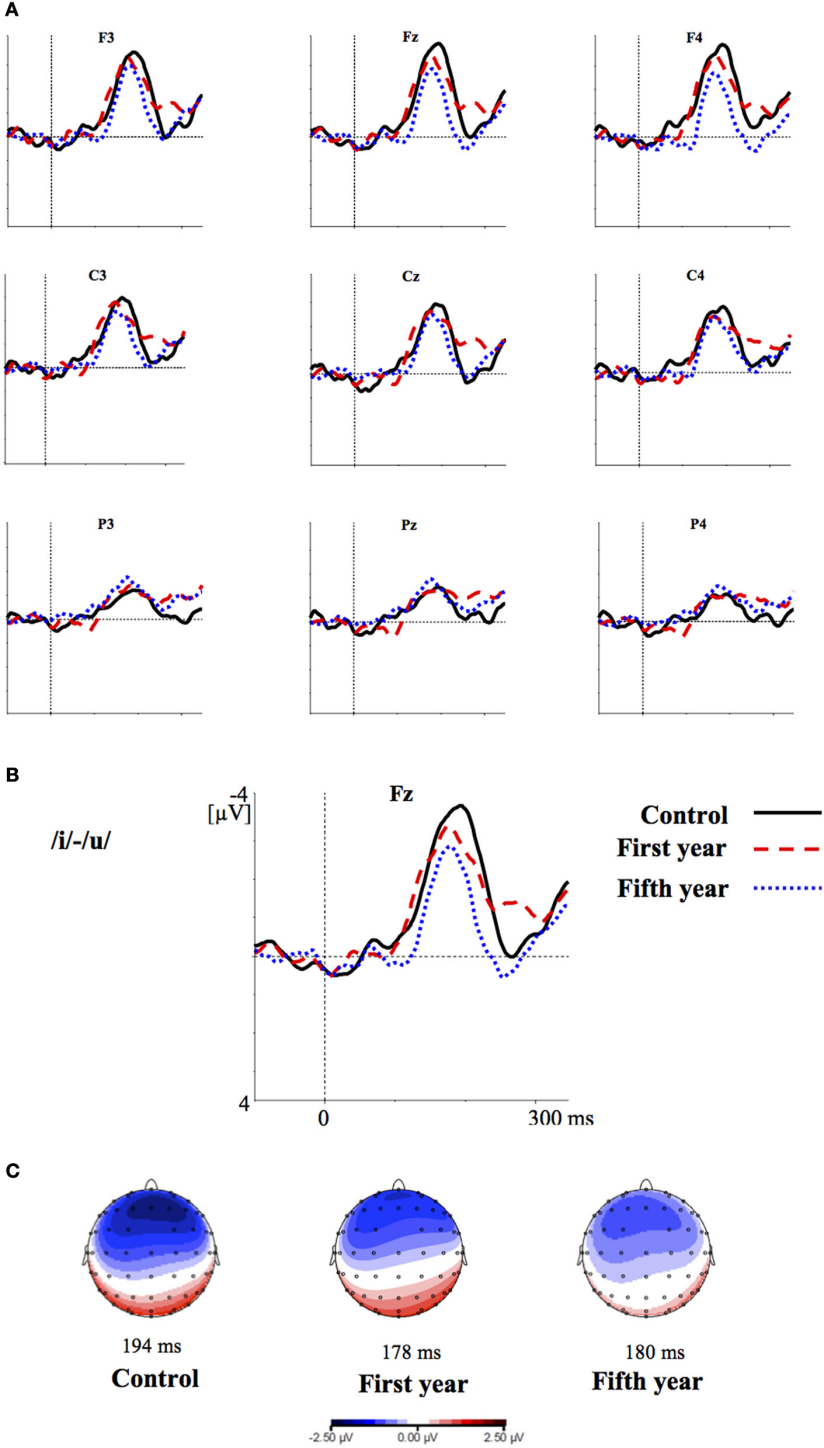
**(A)** Grand-average difference waveforms for the first (blue pointed line) and fifth (red dashed line) year students and the control group (black solid line) in response to the contrast /i/-/u/; **(B)** The grand-average difference waveforms for the three groups at the frontal electrode (Fz) are enlarged; **(C)** Voltage maps for the groups are plotted at the MMN peaks of the grand- average waveforms, referenced to the algebraic mean of the electrodes.

**Figure 3:**
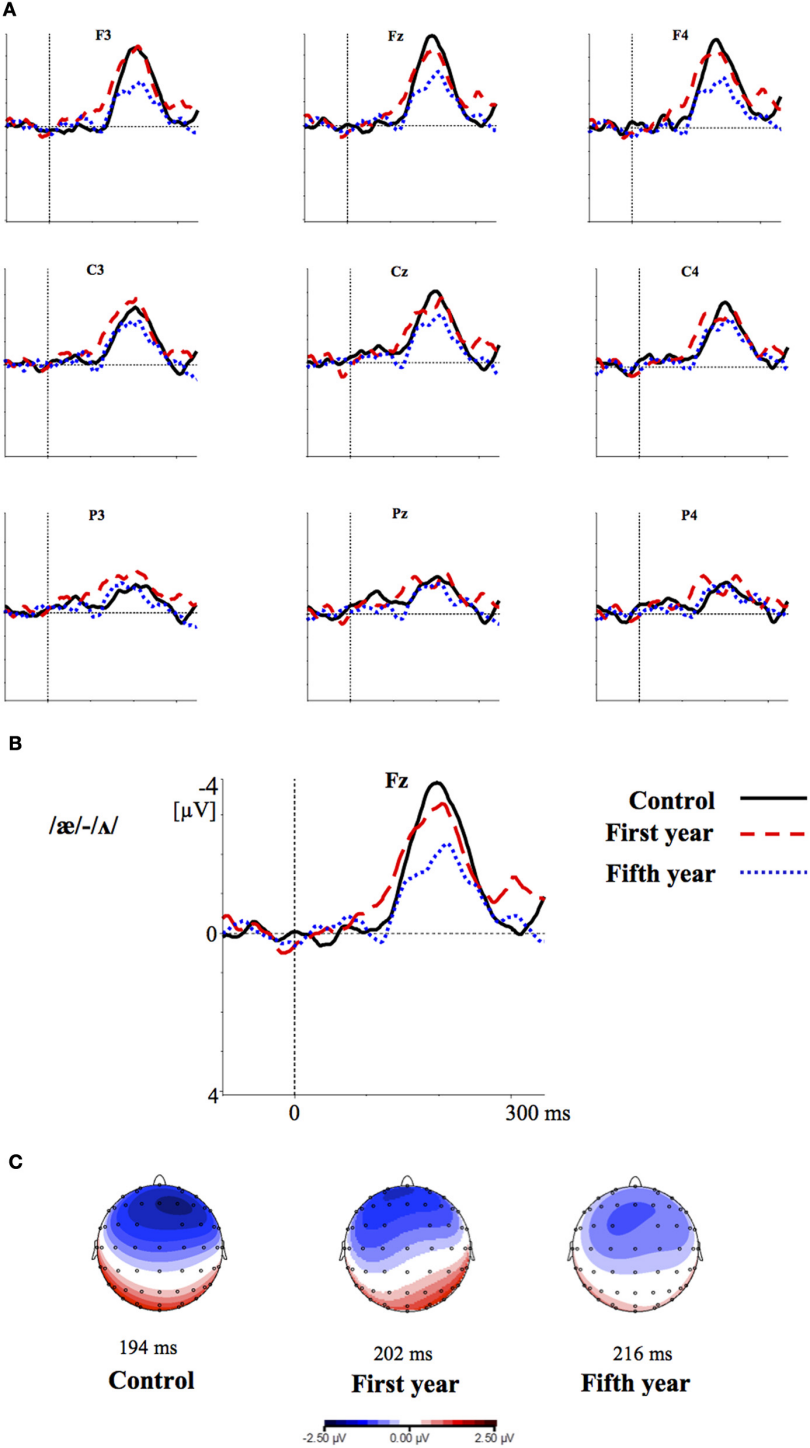
**(A)** Grand-average difference waveforms for the first (blue pointed line) and fifth (red dashed line) year students and the control group (black solid line) in response to the contrast /æ/-/Λ/ **(B)** The grand-average difference waveforms for the three groups at the frontal electrode (Fz) are enlarged; **(C)** Voltage maps for the groups are plotted at the MMN peaks of the grand- average waveforms, referenced to the algebraic mean of the electrodes.

**Figure 4:**
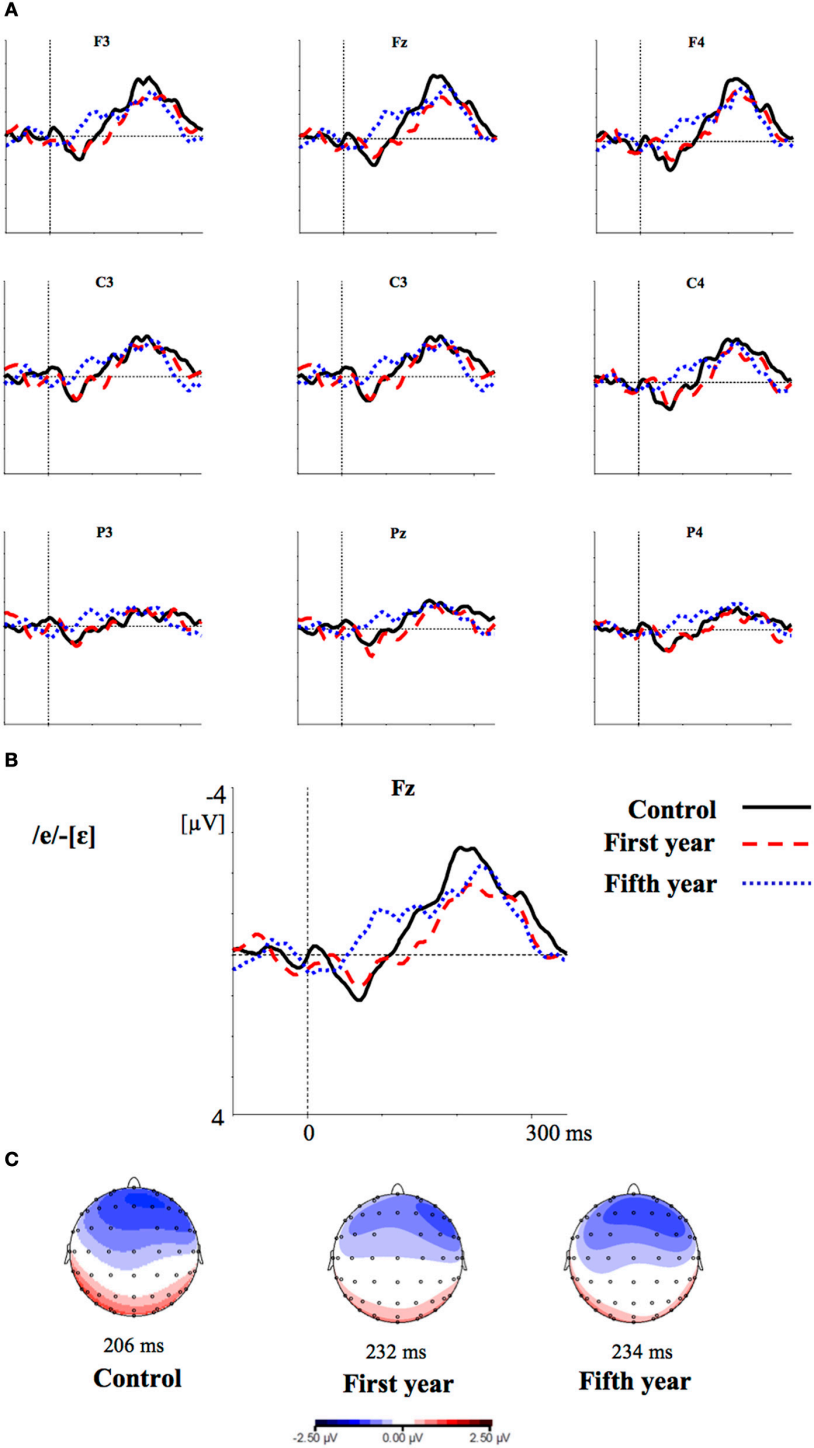
**(A)** Grand-average difference waveforms for the first (blue pointed line) and fifth (red dashed line) year students and the control group (black solid line) in response to the contrast /ε/-[e]; **(B)** The grand-average difference waveforms for the three groups at the frontal electrode (Fz) are enlarged; **(C)** Voltage maps for the groups are plotted at the MMN peaks of the grand- average waveforms, referenced to the algebraic mean of the electrodes.

**Figure 5:**
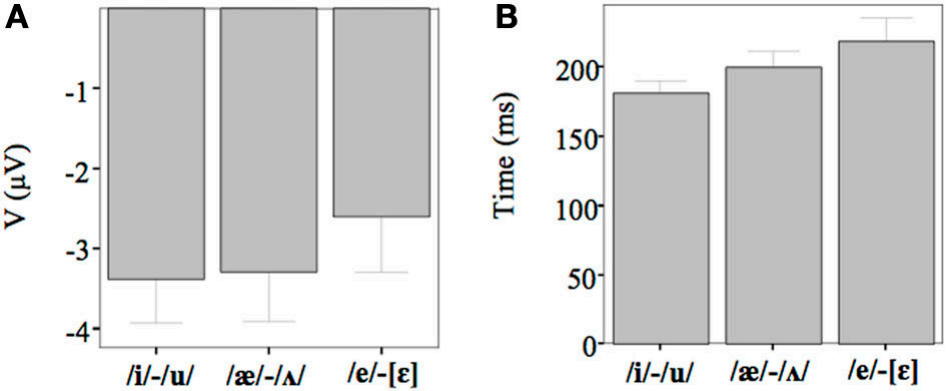
**(A)** The average amplitude (μV) for each contrast. The results are merged since there were no significant differences among the groups. **(B)** The average latency (ms) for each contrast. The results are merged since there were no significant differences among the groups.

Figures 2–4 show the grand-average difference waveforms for all groups and for each stimulus contrast (see also Figure S1 in the Supplementary Material). The mean MMN amplitudes and peak latencies are displayed in Table 5 and Figure 5.

**Table 5:** **The mean MMN amplitudes and peak latencies at Fz**.

**Vowel contrasts**	**I year group**		**V year group**		**Control group**	
	**Amplitude**	**Latency**	**Amplitude**	**Latency**	**Amplitude**	**Latency**
/i/-/u/	3.37 (1.58)	187 (34)	−2.62 (1.45)	176 (13)	−4.26 (1.78)	182 (18)
/æ/-/Λ/	−2.88 (1.00)	185 (21)	−2.73 (1.87)	207 (38)	−3.97 (1.28)	202 (18)
/ε/-[e]	−2.29 (1.36)	230 (35)	−2.39 (1.45)	212 (49)	−3.09 (2.45)	209 (51)

Standard deviations are given in parentheses.

For all conditions and for all groups, we obtained a significant MMN response. In the ANOVA, the MMN amplitude was slightly significantly modulated by Contrast [*F*_(2, 52)_ = 3.02, *p* = 0.05, η^2^_*p*_ = 0.10; this result corresponded to an only marginal significance in the linear mixed-effects model with by-subjects random intercepts where by-stimulus random intercepts and by-subject random slopes for Contrast were tested for inclusion: *F*_(2, 54)_ = 2.9, *p* = 0.07]. The *post-hoc* tests showed that there was a significant difference between the L2 /æ/-/Λ/ and the within-category contrast /ε/-[e] (*p* < 0.05) and a tendency toward a significant difference between /i/-/u/ and the within-category contrast /ε/-[e] (*p* = 0.06). Namely, the within-category contrast /ε/-[e] had the lowest amplitude, while the L2 contrasts /i/-/u/ and /æ/-/Λ/ showed similar amplitudes. The MMN amplitude was also modulated by Frontality [*F*_(2, 52)_ = 112.16, *p* < 0.001, η^2^_*p*_ = 0.81; also replicated in the linear mixed-effects model: *F*_(2, 400)_ = 2.4, *p* < 0.0001] and the *post-hoc* showed that the amplitudes were highest in the frontal area, then in the central and finally in the parietal area. Additionally, we found a modulation of the frontal MMN amplitudes by group expertise with the significant interaction Group × Frontality [*F*_(4, 52)_ = 4.56, *p* < 0.001, η^2^_*p*_ = 0.26; confirmed also in the linear mixed-effects model: *F*_(4, 400)_ = 10.7, *p* < 0.001]. This interaction derived from the larger MMN amplitudes at frontal electrodes to any stimulus found in the control students as compared with the fifth year students (*p* = 0.06).

Moreover, the significant interaction Contrast × Frontality [*F*_(4, 104)_ = 3.38, *p* < 0.05, η^2^_*p*_ = 0.15; this result was replicated in the linear mixed-effects model: *F*_(4, 400)_ = 4, *p* = 0.004] confirmed that in the frontal area the within-category contrast /ε/-[e] had lower amplitudes than /i/-/u/ and /æ/-/Λ/ (/i/-/u/ vs. /ε/-[e]: *p* < 0.05; /æ/-/Λ/ vs. /ε/-[e]: *p* = 0.01; /i/-/u/ vs. /æ/-/Λ/: *p* > 0.05). The typical fronto-central MMN scalp distribution was also confirmed by the significant interaction Frontality × Laterality [*F*_(2, 52)_ = 4.48, *p* = 0.01, η^2^_*p*_ = 0.14; this result was not replicated though in the linear mixed-effect model: *F*_(2, 400)_ = 1.6, *p* = 0.2] and the *post-hoc* showed that this pattern was present in both the right and left hemispheres. The amplitude of the MMN presented a difference in the frontal area only, where it was larger over the right than the left hemisphere (cf. Table 6 for the repeated measures ANOVA results).

**Table 6:** **Degrees of freedom (*df*), *F* and *p* values of the repeated measures ANOVA performed for the MMN amplitudes**.

**Factor**	***df***	***F***	***p*-Value**
Contrast	2, 52	3.02	0.05
Contrast × group	4, 52	0.21	0.93
Frontality	2, 52	112.16	<0.001
Frontality × group	4, 52	4.56	<0.001
Laterality	1, 26	0.06	0.80
Laterality × group	2, 26	0.97	0.39
Contrast × frontality	4, 104	3.38	<0.001
Contrast × frontality × group	8, 104	0.83	0.57
Contrast × laterality	2, 52	0.58	0.56
Contrast × laterality × group	4, 52	0.39	0.81
Frontality × laterality	2, 52	4.48	0.01
Frontality × laterality × group	4, 52	1.36	0.26
Contrast × frontality × laterality	4, 104	0.47	0.75
Contrast × frontality × laterality × group	8, 104	1.68	0.11

The MMN peak latency differed according to the vowel contrasts, as testified by the significant main effect of Contrast [*F*_(2, 52)_ = 10.35, *p* < 0.001, η^2^_*p*_ = 0.28] (cf. Table 7 for all statistical results).

**Table 7:** **Degrees of freedom (*df*), *F* and *p* values of the repeated measures ANOVA performed for the MMN latencies**.

**Term**	***df***	***F***	***p*-Value**
Contrast	2, 52	10.35	<0.001
Contrast × group	4, 52	1.57	0.19

This effect obtained with a general linear model with fixed effects was confirmed also in a linear mixed-effects model of MMN peak latency as a function of Contrast with by-subjects random intercepts where by-stimulus random intercepts for Contrast were tested for inclusion (by-subject random slopes were not included instead, since they did not improve the model fit according to the Bayesian information criteria). Also in this more generalizable mixed-effects model the main effect of Contrast reached significance [*F*_(2, 52)_ = 11.2, *p* < 0.001]. In *post-hoc* tests, the contrasts /i/-/u/ evoked a faster MMN than the contrast /æ/-/Λ/ (*p* = 0.01) and the within-category contrast /ε/-[e] (*p* = 0.000), and in turn the contrast /æ/-/Λ/ evoked a faster MMN than the contrast /ε/-[e] (*p* < 0.05).

## Discussion

This study tested whether the L2 discrimination patterns predicted by the PAM for L2 contrasts are mirrored in the MMN amplitudes and peak latencies to the same contrasts. The behavioral findings suggest that the first and the fifth year students did not differ in their discrimination processes, notwithstanding the different classroom and educational backgrounds. In particular, these two groups of subjects exhibited excellent discrimination of /iː/-/uː/ (belonging to Two-Category assimilation) and moderate to good discrimination of /æ/-/Λ/ (belonging to Uncategorized-Categorized assimilation). The findings obtained in the behavioral experiments are in accordance with the PAM predictions, as the PAM framework foresees excellent discrimination of /iː/-/uː/ and moderate-to-good discrimination of /æ/-/Λ/.

Notably, PAM assimilation types describe the possible perceptive outcomes of first contact with an unfamiliar phonological system and its phonetic patterns. Hence, PAM assimilation types predict how naïve listeners will identify and discriminate non-native phonological contrasts. When a good or an excellent discrimination is predicted, this does not mean that L2 listeners are able to differentiate phonetic and phonological patterns in non-native stimuli, but that they can only easily recognize the acoustic deviations of the unfamiliar phones from their L1 phonemes (Best and Tyler, 2007). According to (Best and Tyler, 2007), this is a starting condition that may or not evolve in the formation of L2 phonetic and phonological categories during the acquisition process, depending on numerous variables: i.e., age of L2 learning, length of residence in an L2-speaking country, gender, formal instruction, motivation, language learning aptitude and amount of native language (L1) use (Piske et al., 2001). The current behavioral findings from both the identification and discrimination tests confirmed in perception those obtained in production by Suter's (1976) seminal work, according to which formal instruction was a factor which did not greatly contribute to the improvement of pronunciation. Suter's study showed that the pronunciation of students does not necessarily improve during their university education. Within the PAM and the SLM framework, supportive evidence, concerning both perception and production, was also behaviorally provided by Simon and D'Hulster (2012). Indeed, L2 university experience in Dutch-speaking learners of English did not have an important effect on their production performance. That is, learners who were almost at the end of their university studies did not produce the English vowel contrast /ε/-/æ/ significantly more native-likely than learners who had only just begun their university studies in English. In parallel, according to PAM, Simon and D'Hulster (2012) found that in perception both inexperienced and experienced learners were able to discriminate the vowel contrast /ε/-/æ/ similarly, since they displayed a Category-Goodness assimilation for which intermediate discrimination is predicted (Best and Tyler, 2007).

In the ERP experiment we introduced a control group of listeners with English knowledge derived only from compulsory school, thus much more inexperienced than the students groups. Furthermore, we introduced a third contrast as control, i.e., the L1 within-category contrast /ε/-[e]. Based on the vowel space of SI, spoken by our subjects (cf. Grimaldi, 2009 and Table 1), we predicted that those two vowels should be perceived as good exemplars of the same native phoneme /ε/. Hence, we expected difficult discrimination for that contrast (Phillips et al., 1995; Dehaene-Lambertz, 1997; Winkler et al., 1999b). Indeed, our electrophysiological results confirmed that in all subjects the two L2 contrasts, /i/-/u/ and /æ/-/Λ/, elicited larger MMN amplitudes than the L1 within-category contrast /ε/-[e] (cf. Table 6). According to PAM predictions, this finding indicates that our subjects discriminated well the two non-native contrasts.

MMN peak latencies, on the other hands, were modulated by the contrast type: the contrast /i/-/u/ elicited a faster MMN than the contrast /æ/-/Λ/ and the within-category contrast /ε/-[e]; in turn, the contrast /æ/-/Λ/ evoked a faster MMN than the contrast /ε/-[e]. This result reflected the acoustic distances between the stimuli (see Table 1), i.e., the smallest between the within-category contrast /ε/-[e] and the largest between the L2 contrast /i/-/u/. As a consequence, the MMN peak latency steadily decreased with increasing acoustic deviation (cf. Näätänen et al., 1997). Actually, the behavioral findings showed that the /i/-/u/ contrast is better discriminated than the /æ/-/Λ/ contrast. So, such fine mirroring of the MMN peak latencies to the behavioral discrimination performances suggests that the perceptual processes manifested by our subjects are influenced by stimulus representations containing mainly auditory (sensory) information.

Furthermore, the MMN peaked at frontal electrodes, was minimal over supra-temporal regions, and was right lateralized. This can shed further light on the nature of the perceptual processes of our subjects (cf. Näätänen et al., 1993; Rinne et al., 2000; Deouell, 2007). Indeed, the MMN generators are usually left lateralized over supra-temporal regions for speech stimuli, whereas the acoustical MMN is bilaterally generated, suggesting that the neural phoneme traces are located in the left auditory cortex (Näätänen et al., 1997; Rinne et al., 1997; Shestakova et al., 2002; Pulvermüller et al., 2003; Shtyrov et al., 2005; see Näätänen et al., 2007 for a discussion). Consequently, the similarity in MMN amplitudes between the groups and the predominant frontal right hemispheric activation suggest a discrimination of auditory sensory information rather than permanent phoneme traces.

Overall, these results confirmed our view based on PAM predictions, namely that both our student groups responded to L2 contrasts as they assimilate them to L1 phonemes, similarly to L2 naïve listeners. If native L2 perceptual abilities had emerged, we would have found significant differences in the MMN amplitude and peak latency responses between the three groups, which was not the case. However, we did find a slight difference in the MMN topography between the groups, although irrespective of the stimulus category: in the frontal electrodes the control group showed more negative MMN amplitudes than the fifth year group of students (Figures 2–4). This effect is most likely deriving from the overlap of the attention-related N2b component on the MMN response (Näätänen, 1992; Escera et al., 1998, 2000), so that the alternating effect of the L2 standard and deviant stimuli produced an attention-modulated neural processing in the less experienced subjects than in the ones more experienced with those speech sounds in general (Näätänen, 1990; Sussman et al., 1998). However, this effect was observed for all stimuli and not modulated by the sound category; hence, is not alone sufficient to claim for neuroplasticity to L2 sounds in the student groups.

Our findings suggest that the amount and the quality of classroom inputs received by our students might be insufficient to form long-term traces of the L2 sounds in their auditory cortex, as indexed by the MMN. This picture is consistent with earlier studies on Finnish children participating in English immersion education and on advanced adult classroom Finnish learners of English (Peltola et al., 2003, 2007) where no MMN traces were found for the development of a new L2 vowel category. Also, the same scenario emerged in studies on limited passive training (Dobel et al., 2009) where MEG data showed that L1 phonemic categories are powerful attractors in that they absorb the non-native stimulus, which is a considerable stumbling block on the path to the mastery of non-native contrasts. Based on these findings, the authors proposed that the maturation of new native-like memory traces is associated with the authenticity of the learning context. However, none of these studies have tested these processes within a theoretical framework on L2 speech learning in adulthood.

## Conclusions and implications for future works

Our study for the first time provides an electrophysiological confirmation of the PAM predictions. Specifically, our results confirm that the PAM framework is able to make predictions on non-native speech perception by L2 listeners who have not actively learned an L2 to achieve functional, communicative goals and that within this typology of learners one has to include L2 classroom learners (Best and Tyler, 2007: 16). Actually, foreign language acquisition usually happens in a pervasive L1 setting (where L2 pronunciation receives little attention) and does not extend much outside the classroom: it often employs formal instruction on lexical and grammatical information and lacks intensive perceptual and pronunciation training (Best and Tyler, 2007). When spoken in the classroom, the L2 is often uttered by L1-accented teachers or, at best, by speakers from diverse L2 varieties, which interferes with perception even for native listeners of the L2 (Bundgaard-Nielsen and Bohn, 2004). Thus, foreign language acquisition is a fairly impoverished context for L2 learning. Indeed, starting from the Suter's (1976) work, behavioral studies examining the influence of formal instruction on the acquisition of L2 foreign perception and production skills have not produced favorable results for language teachers (Flege et al., 1995). The amount of formal inputs received by L2 students has been shown to have a rather limited or null influence, except for the case in which specific training in the perception and production of L2 sounds or a substantial amount of high-quality input over a period of many years is administered (see Piske et al., 2001; Simon and D'Hulster, 2012, and the literature within cited). Thereby, we confirmed and extended the findings of previous behavioral studies (Flege and Fletcher, 1992; Flege, 1995; Flege et al., 1999) in neurally showing that long-term L2 language classroom has no influence on degree of L2 perception and foreign accent. Further studies might, however, utilize novel methods of signal processing to investigate whether differences in neural processing depending on classroom learning might be hidden in narrow EEG frequency bands or in trial-to-trial variations or in corticocortical transfer of information (e.g., Choi et al., 2013; Lieder et al., 2013), which could not be detected with the conventional approach adopted here.

Overall, this and earlier studies support the hypothesis that students in a foreign language classroom should particularly benefit from learning environments only where: (i) receive a focused amount of high-quality input from L2 native teachers; (ii) use pervasively the L2 to achieve functional and communicative goals; and (iii) receive intensive training in the perception and production of L2 sounds in order to reactivate neuroplasticity of auditory cortex (see the issues and studies discussed in Piske, 2007). In fact, recent behavioral and neurophysiological studies (Kraus et al., 1995; Pisoni and Lively, 1995; Tremblay et al., 1997, 1998; Tremblay and Kraus, 2002; Iverson et al., 2005; Ylinen et al., 2009; Zhang et al., 2009) suggest that the sensory resolution of phonetic features can be improved by targeted training, even in adults, and new phonetic representations may be stably developed.

### Conflict of interest statement

The authors declare that the research was conducted in the absence of any commercial or financial relationships that could be construed as a potential conflict of interest.

## Supplementary material

Figure S1 **Power spectral density curves representing the EEG spectrogram recorded at the channel Oz for each subject and the three experimental contrasts**. To plot the curves, the Fourier Transform has been computed over the whole recording of the EEG time series for each subject and condition by using the function Matplotlib in Matlab environment.
