# Supplementary figures and images for: Assimilation of L2 vowels to L1 phonemes governs L2 learning in adulthood: a behavioral and ERP study
Source: Front Hum Neurosci. 2014 May 14;8:279. doi: 10.3389/fnhum.2014.00279 (PMC4030201; doi:10.3389/fnhum.2014.00279)

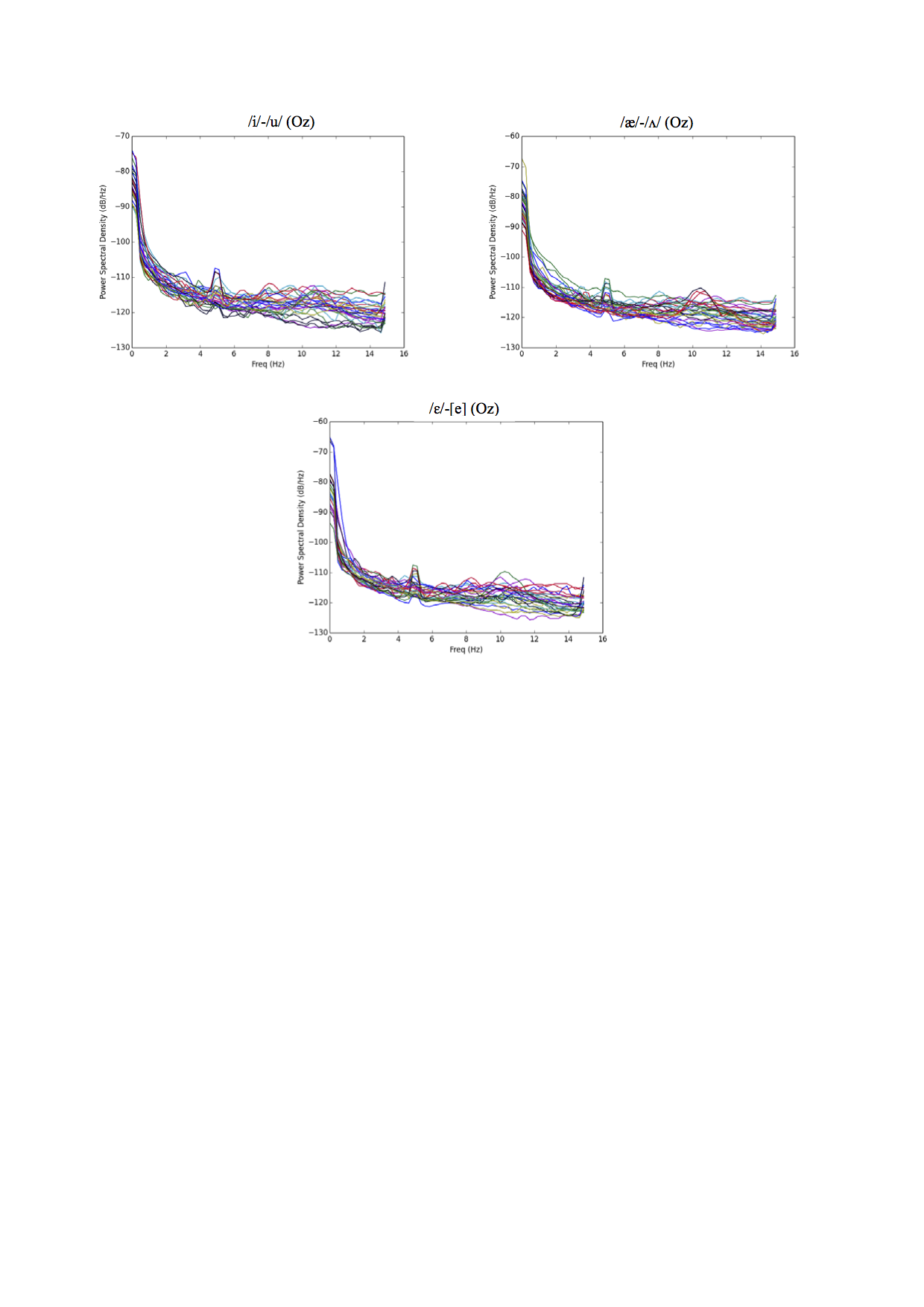

Supplement: Figure S1 — Power spectral density curves representing the EEG spectrogram recorded at the channel Oz for each subject and the three experimental contrasts. To plot the curves, the Fourier Transform has been computed over the whole recording of the EEG time series for each subject and condition by using the function Matplotlib in Matlab environment. [file Presentation1.ZIP › 63751__Supplemental Figure_1.TIFF]
